# Plasmonic Sensing and Switches Enriched by Tailorable Multiple Fano Resonances in Rotational Misalignment Metasurfaces

**DOI:** 10.3390/nano12234226

**Published:** 2022-11-28

**Authors:** Xiaofeng Xu, Xiao-Qing Luo, Qinke Liu, Yan Li, Weihua Zhu, Zhiyong Chen, Wuming Liu, Xin-Lin Wang

**Affiliations:** 1 Hunan Province Key Laboratory for Ultra-Fast Micro/Nano Technology and Advanced Laser Manufacture, School of Electrical Engineering, University of South China, Hengyang 421001, China; 2 School of Nuclear Science and Technology, University of South China, Hengyang 421001, China; 3 Beĳing National Laboratory for Condensed Matter Physics, Institute of Physics, Chinese Academy of Sciences, Beĳing 100190, China; 4 School of Mechanical Engineering, University of South China, Hengyang 421001, China

**Keywords:** Fano resonance, metasurface, plasmonic sensing, plasmonic switch

## Abstract

Fano resonances that feature strong field enhancement in the narrowband range have motivated extensive studies of light–matter interactions in plasmonic nanomaterials. Optical metasurfaces that are subject to different mirror symmetries have been dedicated to achieving nanoscale light manipulation via plasmonic Fano resonances, thus enabling advantages for high-sensitivity optical sensing and optical switches. Here, we investigate the plasmonic sensing and switches enriched by tailorable multiple Fano resonances that undergo in-plane mirror symmetry or asymmetry in a hybrid rotational misalignment metasurface, which consists of periodic metallic arrays with concentric C-shaped- and circular-ring-aperture unit cells. We found that the plasmonic double Fano resonances can be realized by undergoing mirror symmetry along the X-axis. The plasmonic multiple Fano resonances can be tailored by adjusting the level of the mirror asymmetry along the Z-axis. Moreover, the Fano-resonance-based plasmonic sensing that suffer from mirror symmetry or asymmetry can be implemented by changing the related structural parameters of the unit cells. The passive dual-wavelength plasmonic switches of specific polarization can be achieved within mirror symmetry and asymmetry. These results could entail benefits for metasurface-based devices, which are also used in sensing, beam-splitter, and optical communication systems.

## 1. Introduction

Metamaterials that are artificial electromagnetic media have attracted lots of interest owing to their unprecedented optical properties beyond natural materials, thus exhibiting some special electromagnetic responses that include negative-index media [1], zero-index materials [2], and so on [3]. The optical metasurfaces that are two-dimensional metamaterials, which are composed of ultrathin thicknesses and periodic sub-wavelength polarizable unit cells, can be designed to be compatible with traditional semiconductor fabrication techniques [4]. In this sense, optical metasurfaces can be optimized to manipulate the phase, amplitude, and polarization of the wave owing to their ability to locally control geometric parameters [5]. In comparison to the natural materials, the optical metasurfaces can showcase the enhanced and engineered optical responses by their lack of rotational symmetry or in-plane mirror-symmetry [6,7]. As a consequence, there have been enormous efforts devoted to achieving high-sensitivity optical sensing [8], metalens [9], optical switching [10], etc., in optical metasurfaces.

Plasmonic Fano resonance (FR) generated by the interaction between the broad superradiant and narrow subradiant modes [11,12] results in strong field enhancement with a sharp asymmetric spectral profile that is utile for FR-based devices [13]. As in the following, the plasmonic FR promises new avenues for ultrasensitive sensing [14,15,16], optical switches [17], and digital metasurfaces [18]. Compared with a single FR, multiple FRs are more sensitive to structural parameters and the surrounding environment, and have also attracted significant attention due to their further improvement and expansion of functionalities in optical metasurfaces [19,20]. Furthermore, combining two or several nanoparticles into clusters offers a conventional probe of light control and large field enhancement due to their mutual interactions. Considering the different mirror symmetries of the unit cells in the optical metasurfaces, it is not only possible to enable the interaction of high-order dipole modes exhibiting tailorable multiple Fano resonances [21] but also yield new avenues for chiral biosensing [22], circular dichroism [23], and efficient ultrathin circular polarizers [24]. Such mirror symmetries steering in the optical metasurfaces provide a unique characteristic to explore nanoscale light manipulation, which is well resolved in the spectrum via plasmonic FRs.

Introducing an opening angle into the circular-ring-aperture (CRA) unit cells is an efficient way to break the rotational symmetry of the unit cells and elicit the presence of the rotational misalignment metasurface (RMM) with C-shaped-aperture (CSA) unit cells. In this way, it is capable of providing a unique characteristic to explore the manipulation of the optical responses that result from the impact of the in-plane mirror symmetry or asymmetry in an RMM. In this paper, we theoretically propose and numerically demonstrate the near-infrared FRs-based plasmonic sensing and switches empowered by the in-plane mirror symmetry or asymmetry in a hybrid RMM that consists of concentric CSA and CRA unit cells. It is shown that the plasmonic double FRs can be realized by undergoing mirror symmetry along the X-axis, rendering the appearance of double bonding and anti-bonding modes. In particular, by adjusting the level of the mirror asymmetry along the Z-axis, it evolves from a plasmonic FR to double and triple FRs and then back to double FRs as the in-plane mirror symmetry is preserved while simply expanding from FRs to triple FRs when in-plane mirror asymmetry along the Z-axis exists. Moreover, the FRs-based plasmonic sensing that suffer from mirror symmetry or asymmetry when taking on different exponential functions can be implemented by changing the related structural parameters of the unit cells. The passive dual-wavelength plasmonic switches, which are of specific polarization and experience with the in-plane mirror symmetry and asymmetry, can be achieved with high ON/OFF ratios (modulation depth) of 17.53 dB and 10.10 dB (98% and 90%) in the near-infrared region, respectively. The proposed hybrid RMM provides a good alternative strategy for the realization of FRs-based plasmonic sensing and switches, which show great potential for practical applications in metasurface-based devices, sensing, and optical communication systems.

## 2. Structure and Model

**Figure 1:**
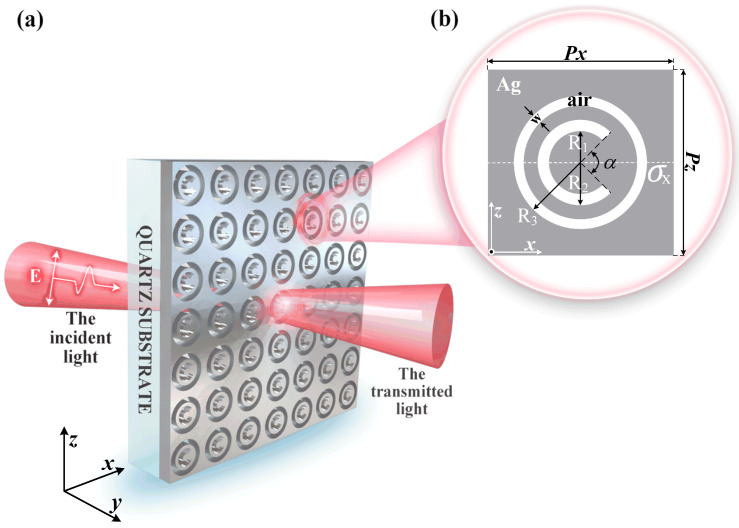
(**a**) Sketch of a hybrid rotational misalignment metasurface composed of periodic silver (Ag) thin-film arrays with concentric C-shaped- (CSA) and circular-ring-aperture (CRA) unit cells. The linearly polarized incident light interacts with the Ag film and propagates from the bottom of the quartz substrate (along the positive Y-direction). (**b**) Structural cross-sectional diagram of a unit cell in the X–Z plane. The gray and white parts represent the Ag film and air, respectively. $R_{1}$ and $R_{2}$ are the inner and outer radii of the CSA structure, respectively. α denotes the opening angle of the CSA structure. *w* and $R_{3}$ depict the width and outer radius of the CRA structure, respectively. $P_{x}$ and $P_{z}$ are the periodic lengths of the Ag array in X and Z directions, respectively.

Herein, we present a hybrid rotational misalignment metasurface (RMM), which comprises periodic hole arrays with concentric C-shaped- (CSA) and circular-ring-aperture (CRA) unit cells, as shown in Figure 1a. The substrate is quartz with a thickness of 225 nm and a dielectric constant of 2.25 [25]. We used a silver (Ag) film with a thickness of 50 nm to establish periodic nano-hole arrays on the quartz substrate [26], owing to its low absorption loss in the visible and near-infrared regions [27]. We defined the dielectric constant of the Ag film by the improved Drude model ($\varepsilon_{r}\left(\omega \right)=\varepsilon_{\infty }-\frac{\omega_{p}^{2}}{\omega^{2}+i\gamma \omega }$), and we set the bulk plasmon frequency ($\omega_{p}$), dielectric constant of infinite frequency ($\varepsilon_{\infty }$), and the damping rate (γ) as $1.3388\times 10^{16}\;\mathrm{rad}/s$, 3.3617, and $7.0759\times 10^{13}\;\mathrm{rad}/s$, respectively. A structural cross-sectional diagram of a unit cell in the X–Z plane is schematically depicted in Figure 1b. The unit cell of the hybrid RMM possesses broken rotational symmetry in the X–Z plane (which is also termed rotational misalignment) and in-plane mirror symmetry along the X-axis $\sigma_{x}$. The X- and Z-direction periodic lengths of the periodic hole arrays are denoted by $P_{x}$ and $P_{z}$, respectively. The gray area represents the Ag film, while the white area delineates the air. $R_{1}$ and $R_{2}$ are the inner and outer radii of the CSA structure, respectively. α denotes the opening angle of the CSA structure. *w* and $R_{3}$ depict the width and outer radius of the CRA structure, respectively. We analyzed the optical transmission characteristics of the hybrid RMM using the three-dimensional finite-difference time-domain approach [28]. The incident light field that is a modulated ultra-short Gaussian pulse interacts with the Ag film and propagates from the bottom of the quartz substrate (along the positive Y-direction) with the central wavelength, width, and center time of the pulse set as 1550 nm, 5 fs, and 16 fs, respectively. To meet the numerical stability conditions, we set the time step and grid size to 5 nm and 8.3 as, respectively. We considered the perfectly matched layers as the boundary conditions on the front and back sides of the sample along the Y-direction. This results in a reflectionless absorbing medium that absorbs without any reflected electromagnetic waves at all frequencies and angles of incidence [29]. Meanwhile, we selected the periodic boundary conditions in the X and Z directions of the unit cell. By the related value and boundary conditions being initialized, alternate samplings of electric and magnetic fields can be executed every half-time step, followed by iterative calculations. We placed a monitoring surface 200 nm above the Ag film to enable the real-time monitoring of the electromagnetic field distribution.

## 3. Results and Discussion

### 3.1. Plasmonic Double Fano Resonances in a Hybrid Rotational Misalignment Metasurface

**Figure 2:**
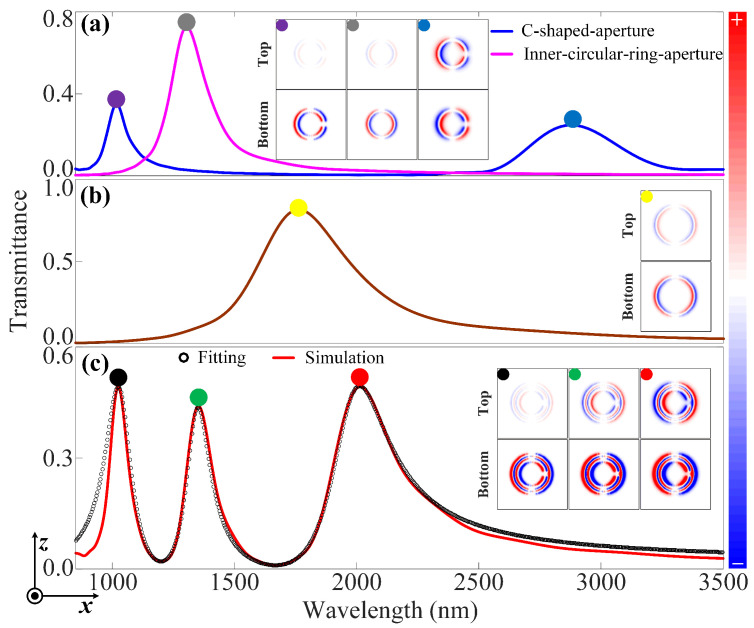
DFRs in a hybrid RMM. The near-infrared transmittance of (**a**) the individual CSA metasurface, (**b**) the individual CRA metasurface, and (**c**) the hybrid RMM. The charge distributions at the labeled wavelengths are shown in the insets, which represent the top (T) panel at the *Ag/air interface* and the bottom (B) panel at the *Ag/quartz interface*. The solid red curve and black circle in (**c**) represent simulation and fitting results, respectively. The geometrical parameters of the unit cell of these metasurfaces are given as follows: $P_{x}$ = $P_{z}$ = 600 nm, $R_{1}$ = 100 nm, $R_{2}$ = 135 nm, α = 20°, *w* = 25 nm, and $R_{3}$ = 175 nm.

Here, we study the near-infrared plasmonic double Fano resonances (DFRs) undergoing in-plane mirror symmetry $\sigma_{x}$ in a hybrid RMM, which is composed of concentrical CSA and CRA unit cells (see Figure 2c). The polarization direction of the incident light is along the X-axis. The unit cell periods of the individual CSA, individual CRA, and hybrid metasurfaces are all set to 600 nm. The inner ($R_{1}$) and outer ($R_{2}$) radii, as well as the opening angle α of the individual CSA and hybrid metasurfaces are set to 100 nm, 130 nm, and 20°, respectively. The outer radius ($R_{3}$) and the width (*w*) of individual CRA and hybrid metasurfaces are set to 175 nm and 25 nm, respectively.

It has already been demonstrated that a metasurface consisting of the CRA unit cells exhibits a polarization-independent transmittance spectrum. It should be noted that a small split of the CRA unit cell evolves to the CSA unit cell, leading to the formation of an RMM. The transmittance spectrum of the individual inner-circular-ring-aperture (ICRA) metasurface (see the pink curve in Figure 2a) shows that the presence of an asymmetric peak is caused by the interaction between the electric dipole ($D_{\mathrm{ICRA}}$) and quadrupole ($Q_{\mathrm{ICRA}}$) modes, giving rise to the pure bonding mode that can be seen as the selective excitation caused by incident light with specific polarization [30]. In contrast, a new peak (see the blue curve in Figure 2a) presents at the transmittance spectrum of the individual CSA metasurface that owns the same parameters as the individual ICRA metasurface, indicating the realization of plasmonic FR. The plasmonic FR originates from the interaction between the electric dipole ($D_{\mathrm{CSA}}$) and quadrupole ($Q_{\mathrm{CSA}}$) modes, thereby inducing the two new hybridized bonding ($D_{\mathrm{CSA}}+Q_{\mathrm{CSA}}$) and anti-bonding ($D_{\mathrm{CSA}}-Q_{\mathrm{CSA}}$) modes. Meanwhile, as shown in the inset of Figure 2a, the charge distributions of the CSA unit cell at the related wavelengths are presented. Therein, the top panel shows the charge distribution at the *Ag/air* interface, while the bottom panel shows the charge distribution at the *Ag/quartz* interface. The charge distributions of the CSA unit cell of the top panel, as shown in the purple dot in Figure 2a, are opposite to the bottom panel, with most of the charges being located at the latter. Furthermore, the charge distributions of the top panel, as shown in the dark blue dot in Figure 2a, are also opposite to the bottom panel; however, those charges are identically distributed in the two panels. For an individual CRA metasurface with larger inner and outer radii, as shown in Figure 2b, similar conclusions can be obtained, while the latter possesses a wider line width and stronger Purcell effect. Likewise, with respect to the charge distribution of the CRA unit cell, as shown in the yellow dot in Figure 2b, the charge properties of the top panel are different from the bottom panel; however, they have nearly identical charge distributions. These behaviors reveal the presence of a pure bonding mode that results from the selective excitation that is due to the incident light with specific polarization. Even the hybridized plasmonic wave functions contain an admixture of $D_{\mathrm{CRA}}$ and $Q_{\mathrm{CRA}}$ modes, and for simplicity, the pure bonding mode can be considered as the effective electric dipolar mode ($D_{\mathrm{CRA}}$) [31]. As shown in Figure 2c, when there is a spectral overlap of transmittance spectra between the bonding ($D_{\mathrm{CSA}}+Q_{\mathrm{CSA}}$) mode of the individual CSA metasurface and the effective electric dipolar mode ($D_{\mathrm{CRA}}$) of the individual CRA metasurface, this can elicit the appearance of two new hybridized *double bonding* ($D_{\mathrm{CSA}}+Q_{\mathrm{CSA}}+D_{\mathrm{CRA}}$) and *anti-bonding* ($D_{\mathrm{CSA}}+Q_{\mathrm{CSA}}-D_{\mathrm{CRA}}$) modes in the hybrid RMM. Furthermore, the degenerate coupling that occurs in the transmittance spectrum between the anti-bonding mode ($D_{\mathrm{CSA}}-Q_{\mathrm{CSA}}$) and the electric dipolar mode ($D_{\mathrm{CRA}}$) directly brings about the occurrence of the *pure double anti-bonding mode* ($D_{\mathrm{CRA}}-D_{\mathrm{CSA}}+Q_{\mathrm{CSA}}$). In brief, the near-infrared plasmonic DFRs can be realized in the hybrid RMM when the system is subjected to in-plane mirror symmetry $\sigma_{x}$ [32]. These behaviors can also be evidenced by the charge distributions in the right inset of Figure 2c.

To gain a better understanding of the underlying physical mechanism of the plasmonic DFRs in our scheme, the multimode interference coupled-mode theory (MICMT) can be used to validate the numerical simulation. It is known to all that, in the case of multiple resonant modes coupling, the coupling phases of different resonant modes that correlate with each other have a significant impact on their transmittance spectra. The MICMT is an effective tool for studying plasmonic FR phenomena, particularly multiple FRs in the hybrid metasurface. In terms of the single-mode–coupled-mode theory, the MICMT fundamental equations with coupling phases can be written as [10,33],

(1) $$\begin{array}{cc} S_{n,1}^{I} & =\gamma_{n1}e^{i\varphi_{n1}}S_{1}^{I}, \end{array}$$

(2) $$\begin{array}{cc} S_{n,2}^{I} & =\gamma_{n2}e^{i\varphi_{n2}}S_{2}^{I}, \end{array}$$

(3) $$\begin{array}{cc} \frac{da_{n}}{dt} & =\left(-i\omega_{n}-\sum_{j=0}^{2}\frac{1}{\tau_{nj}}\right)a_{n}+\kappa_{n1}S_{1}^{I}+\kappa_{n2}S_{2}^{I}, \end{array}$$

(4) $$\begin{array}{cc} S_{1}^{O} & =-S_{1}^{I}+\sum_{n}\kappa_{n1}^{*}a_{n},\;\kappa_{n1}=\sqrt{\frac{2}{\tau_{n1}}}e^{i\theta_{n1}}, \end{array}$$

(5) $$\begin{array}{cc} S_{2}^{O} & =-S_{2}^{I}+\sum_{n}\kappa_{n2}^{*}a_{n},\;\kappa_{n2}=\sqrt{\frac{2}{\tau_{n2}}}e^{i(\theta_{n1}-\phi_{n})}, \end{array}$$

where $S_{j}^{I(O)}\left(j=1,2\right)$ indicate the field amplitudes for the input (output) ports of 1 and 2, respectively. $\gamma_{nj}e^{i\varphi_{nj}}\left(j=1,2\right)$ are the normalized coefficients with $\gamma_{nj}$ being set as 1. $a_{n}$ is the field amplitude of the *n*th resonant mode. $\omega_{n}$ and $\tau_{n0}$ denote the resonant frequency and the decay time of internal loss of the *n*th resonant mode, respectively. $\tau_{n1}$ and $\tau_{n2}$ represent the decay time of the coupling between the *n*th resonant mode and the environment, respectively. $\kappa_{nj}$ and $\theta_{nj}$ (j=1,2) are the coupling coefficients and phases of the *n*th resonant mode, respectively. $\kappa_{nj}^{*}$ (j=1,2) are the related complex conjugate parts of the coupling coefficients. $\phi_{n}$ is the phase difference between the output and input ports of the *n*th resonant mode.

As in the single-input case, i.e., $S_{2}^{I}=0$, the transmittance of the hybrid metasurface can be given as:

(6) $$T=|\frac{S_{2}^{O}}{S_{1}^{I}}{|}^{2}=|\sum_{n=1}^{N}\frac{2e^{i\varphi_{n}}}{-i\left(\omega -\omega_{n}\right)\tau_{n}+2+\frac{\tau_{n}}{\tau_{n0}}}{|}^{2},$$

where $\varphi_{n}$ is the total coupling phase difference for the double bonding ($D_{\mathrm{CSA}}+Q_{\mathrm{CSA}}+D_{\mathrm{CRA}}$), double anti-bonding ($D_{\mathrm{CSA}}+Q_{\mathrm{CSA}}-D_{\mathrm{CRA}}$) and pure double anti-bonding ($D_{\mathrm{CRA}}-D_{\mathrm{CSA}}+Q_{\mathrm{CSA}}$) modes. Using the MICMT to fit the transmittance spectrum of the hybrid RMM, the analytical result (see the black circles in Figure 2c) agrees well with the numerical simulation (see the red solid curve in Figure 2c). The following are the fitting parameters of the DFRs in the hybrid RMM: N=3, $\omega_{1}=2.8416\times 10^{14}\;\mathrm{rad}/s$, $\omega_{2}=2.2044\times 10^{14}\;\mathrm{rad}/s$, $\omega_{3}=1.5065\times 10^{14}\;\mathrm{rad}/s$, $\tau_{1}=160\;\mathrm{fs}$, $\tau_{2}=320\;\mathrm{fs}$, $\tau_{3}=284.6\;\mathrm{fs}$, $\tau_{10}=181\;\mathrm{fs}$, $\tau_{20}=280\;\mathrm{fs}$, $\tau_{30}=285\;\mathrm{fs}$, $\varphi_{1}=0.05$, $\varphi_{2}=0.3$, and $\varphi_{3}=0.35$.

Considering the actual experimental conditions, the periodic metallic hole arrays can be readily fabricated using bottom-up methods. In this way, first, a 50 nm-thick Ag film is deposited on the quartz substrate (225 nm thickness) via radio frequency (RF) sputtering [34,35]. In order to tailor the unit cells with the required geometric configurations of the Ag film, a focused ion beam system can be used to generate symmetrical nanoscale holes with sub-5 nm diameters, thus forming arrays with multiple nanoscale holes [36]. The periodic metallic hole arrays are subsequently milled by using a focused ion beam with a beam current of 750 pA for the individual CSA metasurface, the individual CRA metasurface, and the hybrid RMM. Those metasurfaces are illuminated with a modulated ultra-short Gaussian pulse, where the polarization direction of the incident light can be tuned by using an electromagnetic modulator. Finally, a home-built optical system used for the measurement of the transmission spectra can be applied to collect the transmitted signal through a spectrometer with a charge-coupled device detector [23]. Herein, the Gaussian pulse can be focused on the metasurfaces by one of the near-infrared objective lenses, while the transmitted light is collected by the other near-infrared objective lens. As a consequence, the proposed metasurfaces can be fabricated, and the tailorable plasmonic FRs can be observed in the experiment.

### 3.2. The Tailorable Plasmonic Multiple FRs with In-Plane Mirror Symmetry or Mirror Asymmetry

**Figure 3:**
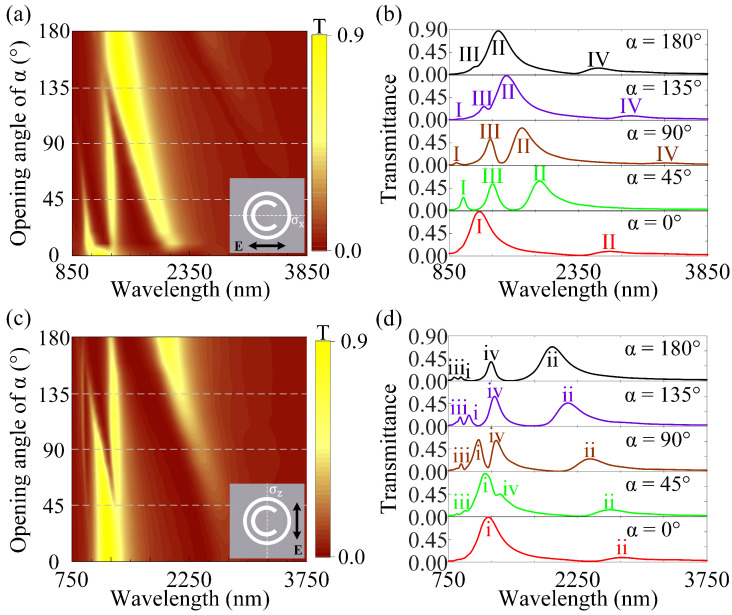
The tailorable multiple FRs with in-plane mirror symmetry or asymmetry in the hybrid RMM. (**a**) The transmittance spectrum is a function of the wavelength and opening angle of α for the hybrid RMM with the incident light being polarized along the X-axis. (**b**) The cross-sections of (**a**) with varying opening angles of α. (**c**) The transmittance spectrum is a function of the wavelength and opening angle of α for the hybrid RMM with the incident light being polarized along the Z-axis. (**d**) are the cross-sections of (**c**) with varying opening angles of α. The other parameters are the same as in Figure 2.

By varying the opening angle α in the hybrid RMM, the in-plane mirror symmetry along the X-axis is preserved, while the in-plane mirror symmetry along the Z-axis is broken (which is also termed as mirror asymmetry) [37]. Next, we study the tailorable plasmonic multiple FRs in the hybrid RMM with in-plane mirror symmetry or asymmetry along the X- and Z-axes. As shown in Figure 3a, for the incident light polarized along the X-axis, the transmittance spectra evolve from plasmonic FR to DFRs and triple FRs and then back to DFRs by varying the opening angle α in the hybrid RMM. Simultaneously, as shown in Figure 3b, the transmission intensity and linewidth of Peak-I and Peak-III decrease gradually, whereas that of Peak-II and Peak-IV increase successively as the opening angle α increases from 0° to 180°. It has already been demonstrated that when the in-plane mirror symmetry is broken in one of the axes, it is possible to present the new resonance peaks and dips near the original symmetric ones [38]. For the incident light that is polarized along the Z-axis, as shown in Figure 3c, the transmittance spectra can simply expand from plasmonic FR to triple FRs by varying the opening angle α in the hybrid RMM. Meanwhile, as shown in Figure 3d, the transmission intensity and linewidth of Peak-II and Peak-IV (Peak-I) gradually increase (then decrease), while that of Peak-III first increase and then decrease as the opening angle α varies from 0° to 180°. In brief, the tailorable plasmonic multiple FRs that experience in-plane mirror symmetry or asymmetry may have promise for potential applications in plasmonic sensing [39,40], plasmonic optical switches [41,42], and digital metasurfaces [43], as well as metalenses [9].

### 3.3. Plasmonic Sensing with In-Plane Mirror Symmetry and Mirror Asymmetry

**Figure 4:**
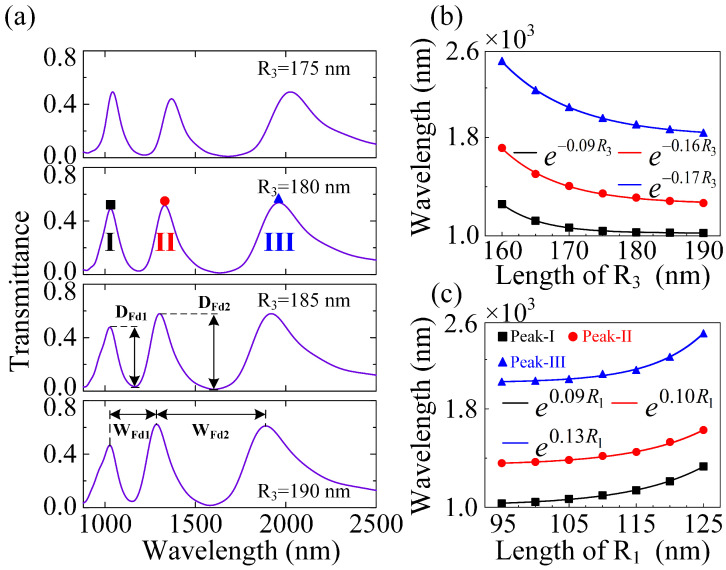
DFRs-based plasmonic sensing with in-plane mirror symmetry $\sigma_{x}$. (**a**) The transmittance spectra for the hybrid RMM with different outer radii ($R_{3}$) of the CRA unit cells, where the incident light is polarized along the X-axis. (**b**) The resonance center wavelengths of the three peaks of the plasmonic DFRs in the hybrid RMM as three different exponential decrease functions of the outer radius ($R_{3}$) of the CRA unit cells. (**c**) The resonance center wavelengths of the three peaks of the plasmonic DFRs in the hybrid RMM as three different exponential increase functions of the inner radius ($R_{1}$) of the CSA unit cells. The other parameters are the same as Figure 2.

It is well understood that FRs-based plasmonic sensing have attracted tremendous attention in the nanophotonics community owing to their strong interactions with the dielectric environment [44,45]. Additionally, it has been proven that optical metasurfaces show great potential in plasmonic sensing for their advantages in flexibly controlling light–matter interactions [46]. The manipulation of mirror symmetry in optical metasurfaces has contributed to a range of distinct structures and responses [47]. The exponential red- or blue-shift of the wavelength of the resonance modes has greatly enriched our study of plasmonic sensing in the near-infrared region [48]. Following that, considering the resonance response of the hybrid metasurface is significantly influenced by the geometrical parameters of the unit cells, one can modulate the DFR-based plasmonic sensing by adjusting them in the hybrid RMM. As shown in Figure 4a, the $W_{F_{\mathrm{di}}}$ and $D_{F_{\mathrm{di}}}$ (i=1,2) are defined as the width and depth of the Fano dips, respectively. For the polarization direction of the incident light to be fixed along the X-axis, the in-plane mirror symmetry $\sigma_{x}$ is preserved at this time. As the other parameters are held constant, by varying the outer radius ($R_{3}$) of the CRA unit cells from 160 to 190 nm, the resonance center wavelengths of the three peaks of the plasmonic DFRs (see the color symbols in Figure 4b) are blue-shifted by different exponential decrease functions (see the solid color curves in Figure 4b). Simultaneously, the widths of the Fano dips are gradually narrowing, with $W_{\mathrm{Fd}1}$ getting much narrower than $W_{\mathrm{Fd}2}$. The Fano depth ($D_{\mathrm{Fd}1}$) becomes deeper and deeper, whereas the Fano depth ($D_{\mathrm{Fd}2}$) first increases (as $R_{3}$ ranges from 160 to 180 nm) and then decreases (as $R_{3}$ ranges from 180 to 190 nm). To quantify the sensitivity of the proposed plasmonic sensing, we bring the CSA structure into an effective radius ($\Delta R_{n}=\left(R_{n}-R_{n}^{\min}\right)/R_{n}^{\max}$) to define a shifted coefficient in the resonant wavelength per unit change, thus leading to the sensitivity for plasmonic sensing being defined as Δλ/$\Delta R_{n}$. These behaviors are well fitted by different sine functions, which can also be interpreted as the enhancement of the interaction between CSA and CRA structures. Additionally, the sensitivities of peak-I, peak-II, and peak-III (see the black square, red circle, and blue triangle in Figure 4b) can be obtained as 1590, 3027, and 3946, respectively. Conversely, by only changing the inner radius ($R_{1}$) of the CSA unit cells from 95 to 125 nm, the resonance center wavelengths of the three peaks of the plasmonic DFRs (see the color symbols in Figure 4c) are red-shifted by different exponential increase functions (see the solid color curves in Figure 4c). The two widths of the plasmonic DFRs are continually narrowing, with $W_{\mathrm{Fd}1}$ narrowing faster than $W_{\mathrm{Fd}2}$. The two depths of the plasmonic DFRs first increase (as $R_{1}$ ranges from 95 to 105 nm) and then decrease (as $R_{1}$ ranges from 105 to 125 nm), which are well fitted by different sine functions and can also be ascribed to the weakened intensity of the bonding ($D_{\mathrm{CSA}}+Q_{\mathrm{CSA}}$) and anti-bonding ($D_{\mathrm{CSA}}-Q_{\mathrm{CSA}}$) modes in the hybrid RMM. Additionally, the sensitivities of peak-I, peak-II, and peak-III (see the black square, red circle, and blue triangle in Figure 4c, respectively) can be obtained as 1254, 1125, and 1638, respectively. In short, the DFRs-based plasmonic sensing that suffer from in-plane mirror symmetry $\sigma_{x}$ are more sensitive to the outer radius ($R_{3}$) of the CRA unit cells in the hybrid RMM.

**Figure 5:**
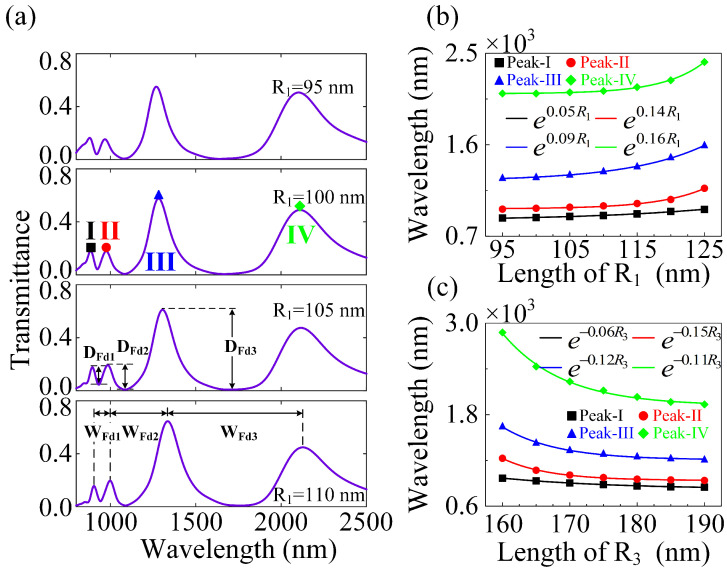
Triple-FRs-based plasmonic sensing with in-plane mirror asymmetry $\sigma_{z}$. (**a**) The transmittance spectra for the hybrid RMM with different inner radii ($R_{1}$) of the CSA unit cells, where the incident light is polarized along the Z-axis. (**b**) The resonance center wavelengths of the four peaks of the triple FRs in the hybrid RMM as four different exponential increase functions of the inner radius ($R_{1}$) of the CSA unit cells. (**c**) The resonance center wavelengths of the four peaks of the triple FRs in the hybrid RMM as four different exponential decrease functions of the outer radius ($R_{3}$) of the CRA unit cells. The other parameters are the same as Figure 2.

With respect to the in-plane mirror asymmetry $\sigma_{z}$, we show that triple-FRs-based plasmonic sensing in the hybrid RMM can be steered by altering the inner radius ($R_{1}$) of the CSA unit cells and outer radius ($R_{3}$) of the CRA unit cells. As shown as in Figure 5a, the $W_{F_{\mathrm{di}}}$ and $D_{F_{\mathrm{di}}}$ (i=1,2,3) are defined as the width and depth of the Fano dips, respectively. Because the polarization direction of the incident light is fixed along the Z-axis, the in-plane mirror symmetry $\sigma_{z}$ is definitely broken. As the other parameters are held constant, by changing the inner radius ($R_{1}$) of the CSA unit cells from 95 to 110 nm, the resonance center wavelengths of the four peaks of the plasmonic triple FRs (see the color symbols in Figure 5b) are red-shifted by different exponential increase functions (see the solid color curves in Figure 5b). Likewise, the two widths of the Fano dips ($W_{\mathrm{Fd}1}$ and $W_{\mathrm{Fd}2}$) are gradually broadening, with $W_{\mathrm{Fd}2}$ getting much broader than $W_{\mathrm{Fd}1}$, and the width of the Fano dip ($W_{\mathrm{Fd}3}$) is slightly narrowing. The two Fano depths ($D_{\mathrm{Fd}1}$ and $D_{\mathrm{Fd}2}$) first increase and then decrease; however, the Fano depth ($D_{\mathrm{Fd}3}$) becomes shallower by changing the inner radius ($R_{1}$) of the CSA unit cells in the hybrid RMM. Moreover, the sensitivities of peak-I, peak-II, peak-III, and peak-IV (see the green diamond, black square, red circle, and blue triangle in Figure 5b, respectively) can be acquired as 354, 854, 1338, and 1292, respectively. On the contrary, by just adjusting the outer radius ($R_{3}$) of the CRA unit cells from 160 to 190 nm, the resonance center wavelengths of the four peaks of the plasmonic triple FRs (see the color symbols in Figure 5c) are blue-shifted by different exponential decrease functions (see the solid color curves in Figure 5c). Therein, the three widths of the plasmonic triple FRs are continually narrowing, with $W_{\mathrm{Fd}3}$ becoming narrower faster than $W_{\mathrm{Fd}1}$ and $W_{\mathrm{Fd}2}$, which can be well fitted by different exponential decrease functions. The two depths of the plasmonic triple FRs ($D_{\mathrm{Fd}1}$ and $D_{\mathrm{Fd}2}$) first increase (as $R_{3}$ ranges from 160 to 175 nm) and then decrease (as $R_{3}$ ranges from 175 to 190 nm), whereas the depth ($D_{\mathrm{Fd}3}$) unceasingly becomes deeper, which can be well fitted by different sine functions. Additionally, the sensitivities of peak-I, peak-II, peak-III, and peak-IV (see the green diamond, black square, red circle and blue triangle in Figure 5c, respectively) can be acquired as 751, 1844, 2761, and 5979, respectively. Consequently, triple-FRs-based plasmonic sensing with in-plane mirror asymmetry along the Z-axis is comparatively susceptible to the inner radius ($R_{1}$) of the CSA unit cells and the outer radius ($R_{3}$) of the CRA unit cells in the hybrid RMM.

### 3.4. Dual-Wavelength Plasmonic Switches with In-Plane Mirror Symmetry and Mirror Asymmetry

**Figure 6:**
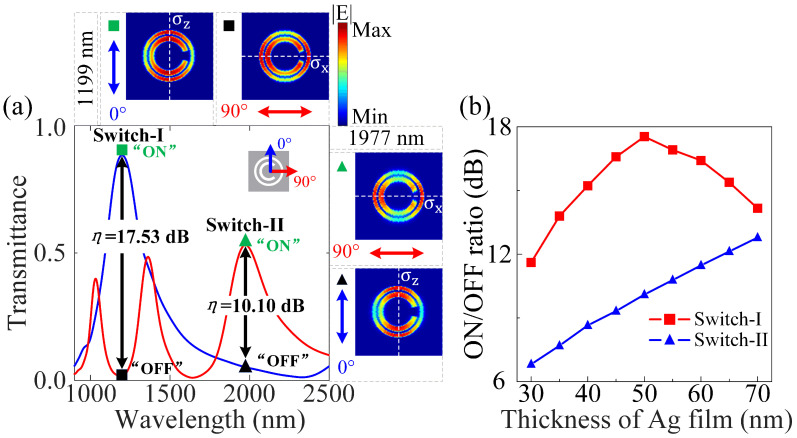
Passive dual-wavelength plasmonic switches with in-plane mirror symmetry and mirror asymmetry along the X- ($\sigma_{x}$) and Z-axes ($\sigma_{z}$), respectively. (**a**) The passive plasmonic switches versus the wavelength with different polarization angles of incident light in the near-infrared region. The high ON/OFF ratios are 17.53 dB and 10.10 dB for the resonance center wavelengths at 1199 nm and 1977 nm, respectively. The corresponding electric field distributions of the hybrid RMM are shown in the insets. (**b**) For the resonance center wavelengths at 1199 nm and 1977 nm, the ON/OFF ratios change with the increase in the thickness of Ag film. The opening angle (α) of the hybrid metasurface is fixed as 30°, with the other parameters being the same as Figure 2.

It has been found that the optical switch that enables the signal to be turned on and off by another controller plays an important role in routing the optical signal in optical communication and information processing [49]. However, the prevailing way of optical switching usually requires an auxiliary power supply and additional control circuits that are physically connected to the metasurface, thus leading to increasing the size of the system and inducing needless cross-talk [50,51]. In our scheme, the maximal and minimal of the transmittance spectra can be performed as the “ON” and “OFF” states of the passive plasmonic switch at the related wavelengths, respectively. For the central wavelengths of 1199 nm and 1977 nm, as shown in Figure 6a, their transmittance spectra can be modulated by regulating the polarization direction of the incident light, accompanied directly with the presence/absence of the in-plane mirror symmetry. The ON/OFF ratio (η) and modulation depth (MD) can be written as [18,52],

(7) $$\begin{array}{cc} \eta & =10\log_{10}\left(\frac{T_{\mathrm{ON}}}{T_{\mathrm{OFF}}}\right), \end{array}$$

(8) $$\begin{array}{cc} \mathrm{MD} & =\left(1-\frac{T_{\mathrm{OFF}}}{T_{\mathrm{ON}}}\right)\times 100\%, \end{array}$$

where $T_{\mathrm{ON}}$ and $T_{\mathrm{OFF}}$ depict the “ON” and “OFF” states at the related wavelengths, respectively.

Herein, we show that the passive plasmonic switches (i.e., Switch-I and Switch-II) can be accomplished in the near-infrared region with the in-plane mirror symmetry along the X- ($\sigma_{x}$) and Z-axes ($\sigma_{z}$), respectively. As shown in Figure 6a, regarding the resonance center wavelength at 1199 nm, the FR-based “ON” (DFRs-based “OFF”) state of the transmittance spectrum can be realized without (with) the in-plane mirror symmetry $\sigma_{z}$ ($\sigma_{x}$) by containing the incident light polarized along the Z-axis (X-axis) and $T_{\mathrm{ON}}=0.8837$ ($T_{\mathrm{OFF}}=0.01562$). Additionally, the η and MD can be calculated as 17.53 dB and 98% by using Equations (7) and (8), respectively. It is of great interest to notice that the resonance center wavelength of Switch-I can be extended to 1352 nm (in the telecom O-band: 1260∼1360 nm) by modulating the related geometric parameters of the hybrid RMM, with the η and MD being 13.32 dB and 95%, respectively. With regard to the resonance center wavelength at 1977 nm, the DFRs-based “ON” (FR-based “OFF”) state of the transmittance spectrum can be realized with the in-plane mirror symmetry $\sigma_{x}$ (mirror asymmetry $\sigma_{z}$), including the incident light polarized along the X-axis (Z-axis) and $T_{\mathrm{ON}}=0.5313$ ($T_{\mathrm{OFF}}=0.05191$). Analogously, the η and MD can be carried out to be 10.10 dB and 90%, respectively. Furthermore, as shown in Figure 6b, for the resonance center wavelength of Switch-I at 1199 nm, by varying the thickness of the Ag film, the ON/OFF ratio (η) first increases and then decreases [53], with a maximal ratio (17.53 dB) existing at an Ag film thickness of 50 nm. For the resonance center wavelength of Switch-II at 1977 nm, the ON/OFF ratio exhibits linearly increasing characteristics by changing the thickness of the Ag film.

## 4. Conclusions

In summary, we have investigated the near-infrared plasmonic Fano resonances for plasmonic sensing and switches by undergoing different in-plane mirror symmetries in a hybrid rotational misalignment metasurfaces that consist of periodic Ag film arrays with concentric CSA and CRA unit cells. It is worth noting that a small split of the CRA unit cell evolves into a CSA unit cell, leading to the formation of a rotational misalignment metasurface, revealing the presence of near-infrared plasmonic Fano resonance originating from the interaction between the electric dipole ($D_{\mathrm{CSA}}$) and quadrupole ($Q_{\mathrm{CSA}}$) modes. The in-plane mirror symmetry $\sigma_{x}$ in the hybrid rotational misalignment metasurface can induce three new hybridized resonance modes from the plasmonic double Fano resonances in the hybrid rotational misalignment metasurface (that is, double bonding ($D_{\mathrm{CSA}}+Q_{\mathrm{CSA}}+D_{\mathrm{CRA}}$), double anti-bonding ($D_{\mathrm{CSA}}+Q_{\mathrm{CSA}}-D_{\mathrm{CRA}}$), and pure double anti-bonding ($D_{\mathrm{CRA}}-D_{\mathrm{CSA}}+Q_{\mathrm{CSA}}$) modes), which also agree well with the MICMT. Moreover, by varying the opening angle of α, the transmittance spectra evolve from Fano resonance to double and triple Fano resonances and then back to double Fano resonances as the in-plane mirror symmetry $\sigma_{x}$ is preserved, while simply expanding from Fano to triple Fano resonances when the in-plane mirror asymmetry $\sigma_{z}$ is broken. Furthermore, by varying the inner radius ($R_{1}$) of the CSA or the outer radius ($R_{3}$) of the CRA unit cells in the hybrid rotational misalignment metasurface, double- and triple-Fano-resonance-based plasmonic sensing can be fulfilled with in-plane mirror symmetry $\sigma_{x}$ and asymmetry $\sigma_{z}$, including the exponential red- or blue-shift of the center wavelength of the resonance modes, respectively. The Fano-resonance-based passive plasmonic switches that suffer from in-plane mirror symmetry and asymmetry can be achieved with high ON/OFF ratios (modulation depth) of 17.53 dB and 10.10 dB (98% and 90%) in the near-infrared region, respectively. The ON/OFF ratios of Switch-I and Switch-II are distinctly susceptible to the thickness of the Ag film.

Furthermore, adjustable double- and triple-Fano-resonance-based plasmonic sensing are conducive to sizing single particles [54], detecting nanometer-scale objects [55], and monitoring and analyzing biomolecular binding events [56]. The Fano-resonance-based passive plasmonic switches that undergo different in-plane mirror symmetries can be realized as compact devices instead of adding further active pump light fields and control circuits in the hybrid rotational misalignment metasurface. Our results are also beneficial to improving optical communication applications according to their arbitrary building blocks and size-dependent spectral scalability.

## Data Availability

The data presented in this study are available on request from the corresponding author.
